# Cloning, mapping and molecular characterization of porcine progesterone receptor membrane component 2 (*PGRMC*2) gene

**DOI:** 10.1590/S1415-47572010005000057

**Published:** 2010-09-01

**Authors:** Congying Chen, Carole Sargent, Claire Quilter, Zhuqing Yang, Jun Ren, Nabeel Affara, Bertram Brenig, Lusheng Huang

**Affiliations:** 1 Key Laboratory for Animal Biotechnology of Jiangxi Province and The Ministry of Agriculture of China, Jiangxi Agricultural University, Nanchang, Jiangxi ProvincePeople's Republic of China; 2 Mammalian Molecular Genetics Group, Division of Cellular and Molecular Pathology, Department of Pathology, University of Cambridge, CambridgeUK; 3 Institute of Veterinary Medicine, Georg-August-University of Goettingen, Burckhardtweg 2, GoettingenGermany

**Keywords:** expression profile, molecular characterization, physical mapping

## Abstract

Progesterone plays an important role in sow reproduction by stimulating classic genomic pathways via nuclear receptors and non-genomic pathways via membrane receptors such a progesterone receptor membrane component 2 *(PGRMC*2). In this work, we used radiation hybrid mapping to assign *PGRMC*2 to pig chromosome 8 and observed that this receptor has two transcripts in pigs. The full-length cDNA of the large transcript is 1858 bp long and contains a 669-bp open reading frame (ORF) encoding a protein of 223 amino acids. The shorter transcript encodes a protein of 170 amino acids. The porcine *PGRMC*2 gene consists of three exons 446 bp, 156 bp and 1259 bp in length. The promoter sequence is GC-rich and lacks a typical TATA box. Several putative cis-regulatory DNA motifs were identified in the 208-bp upstream genomic region. Five single nucleotide polymorphisms (SNPs) were detected in introns* and the 3' UTR. RT-PCR indicated that the *PGRMC*2 gene is expressed ubiquitously in all pig tissues examined.

Progesterone plays an important role in sow reproduction and maternal behavior. In mice, progesterone receptor blockade during late pregnancy leads to abnormal maternal behavior including infanticide (Wang *et al.*, 1995). Progesterone exerts its physiological effects by activating two major signaling pathways, namely the classic genomic pathway and the non-genomic pathway. In the former pathway, the hormone binds to cytosolic receptors and subsequently modulates gene expression, leading to alterations in protein synthesis. In the latter pathway, hormone signaling is mediated by membrane receptors that are still poorly characterized and unrelated to intracellular steroid receptors associated with the genomic pathway (Losel *et al.*, 2003). Gerdes *et al.* (1998) cloned two human putative steroid binding membrane proteins, Hpr6.6 (*PGRMC*1) and Dg6 (*PGRMC*2). In addition, the human genes *PGRMC*1 and *PGRMC*2 that encode progesterone binding membrane proteins have also been cloned and extensively characterized (Bernauer *et al.*, 2001; Losel *et al.*, 2005). The full-length cDNA sequence of the porcine *PGRMC*1 gene from vascular smooth muscle cells has been described (Falkenstein *et al.*, 1996), whereas little is known about the *PGRMC*2 gene in pigs. In this report, we describe the molecular characterization, physical mapping and expression profile of porcine *PGRMC*2.

Two porcine ESTs that shared 95% sequence homology with the human *PGRMC*2 cDNA were identified in the GenBank databa**s**e (GenBank accession nos. BP147690 and DN105047). These EST sequences were used to design primers for porcine *PGRMC*2. The full-length cDNA of *PGRMC*2 obtained by using the reverse transcription polymerase chain reaction (RT-PCR) and rapid amplification of cDNA ends (RACE). RACE was done by using a Smart RACE cDNA amplification kit according to the manufacturers instructions (BD Biosciences Clontech, USA) with nested PCR (see Table 1 for RACE primers). The 5' RACE assay produced two unambiguous fragments of 221 bp and 335 bp, indicating at least two alternative transcripts of *PGRMC*2 in pigs (Figure 1). The 5' RACE and 3' RACE fragments, ESTs, and gap fragment between two ESTs amplified with the P1 primers were assembled online with the CAP3 Sequence Assembly Program to obtain the full-length *PGRMC*2 cDNA. The longer transcript consisted of 1858 bp (GenBank accession no. EU242513). The open reading frame (ORF) of this transcript was 672 bp long and was flanked by a 25 bp 5' UTR and an 1161 bp 3' UTR, as predicted with the online ORF finder tool.

**Figure 1:**
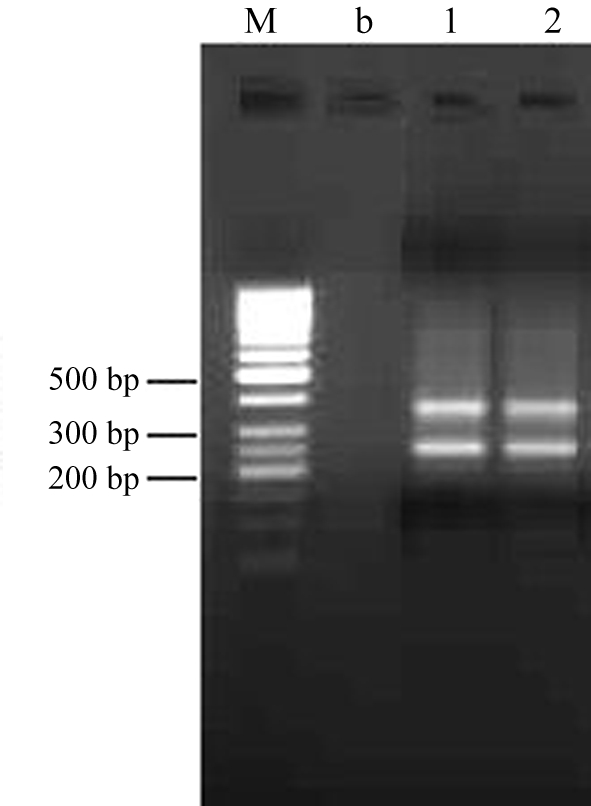
Two transcripts of *PGRMC*2 in porcine hypothalamus and liver revealed by a 5' RACE assay. M: 50 bp DNA ladder marker, b: blank control, 1: hypothalamus, 2: liver.

Further analysis using SMART tools online showed that the ORF encoded a protein of 223 amino acids with a calculated molecular mass of 23.77 kDa and an isoelectric point (pI) of 4.77. The shorter transcript of 1744 bp contained a 70 bp 5' UTR, a 513 bp ORF and a 1161 bp 3' UTR. The two transcripts shared the same exons 2 and 3. The deduced protein encoded by the shorter transcript consisted of 170 amino acids, with a molecular mass and pI of 19.08 kDa and 5.37, respectively. Both of the deduced proteins contained a cytochrome b5-like heme/steriod binding domain composed of 100 amino acids with a 23 amino acid transmembrane domain in the long variant and a signal peptide of 18 amino acids in the short variant. These findings indicated that, as with human *PGRMC*1 and *PGRMC*2 (Mifsud and Bateman, 2002), porcine *PGRMC*2 was also a membrane receptor belonging to the cytochrome b5 superfamily. The cytochrome b5-like heme/steroid binding domain can bind several steroid hormones, including progesterone (100%), testosterone (20%), and cortisol (4%) (Meyer *et al.*, 1996), and may have an important role as a receptor in modulating the effect of steroids in reproduction.

BLAST searches indicated that the predicted porcine *PGRMC*2 sequence shared high identity with orthologs of other mammals, *i.e.*, 97% identity with *Canis familiaris* (GenBank accession no. XP_533292), 96% identity with human (GenBank accession no. NP_006311), bovine (GenBank accession no. XP_613630) and ape (GenBank accession no. XP_517434), 90% identity with mouse (GenBank accession no. XP_992902), and 89% identity with rat (GenBank accession no. NP_001008375).

The 3' UTR contained one consensus polyadenylation signal (AATAAA) and three pentanucleotide (ATTTA) mRNA destabilization motifs.

The *PGRMC*2 genomic structure was initially predicted by alignment of the porcine full-length cDNA sequence with human and mouse *PGRMC*2 genomic DNA sequences (GenBank accession nos. DQ496105 and AC100511). The genomic DNA organization of the porcine *PGRMC*2 gene was similar to that of other mammalian *PGRMC*2 genes in that it consisted of two introns and three exons. Exon 1 was 443 bp and 329 bp long in the long and short transcripts, respectively. Exons 2 and 3 were identical in both transcripts and were 156 bp and 1259 bp long, respectively. The sequences for introns 1 and 2 were obtained by Extensor Hi-Fidelity PCR (ABgene, Surrey, UK) and by primer walking using primers P2, P3 and P4 (Table 1), respectively. The size of intron 1 (~14-15 kb) was similar in human and pig whereas intron 2 was more variable, *i.e.*, 1855 bp in pig compared to 948 bp in the human genome (Genbank accession no. AC096898). The exon/intron boundaries were absolutely conserved among mammals and conformed to the GT/AG splicing rule (GenBank accession no. EU242514). These features indicate that the *PGRMC*2 gene is well conserved in mammalian evolution.

Amplification of the 5' flanking region with primer P5 yielded 5' genomic DNA sequences of 208 bp and 323 bp for the long and short transcripts, respectively. As with the human *PGRMC*2 promoter region (Bernauer *et al.*, 2001), the 5' flanking region of the porcine *PGRMC*2 gene was GC-rich and sequence analysis revealed a CpG island spanning the transcriptional start site of the long transcript. The promoter sequence lacked a TATA box characteristic of housekeeping genes. The *cis*-CCAAT box (the putative CTF/NF-1 binding site) at position -288 bp (the transcriptional start site of the long transcript was marked +1 and is used as the reference point in the positions described below) was conserved in the promoter region of the human and porcine *PGRMC*2 genes. A *glyco* motif (AACGTTAC) and an *RBS* motif (AGGAGG) were also identified at positions -145 to -152 bp and -23 to -28 bp, respectively. There were four Sp1- binding motifs at positions -211 bp, -64 bp, -34 bp and -23 bp. Two more Sp1- binding motifs were present in the 5' promoter region of the short transcript at positions +111 bp and +150 bp. Two T-Ag binding sites (GGGGC) were located at positions -61 bp and -222 bp. One AP-2 binding site was found at -105 bp. There were four GAGA boxes in the promoter region of the porcine *PGRMC*2 gene, at -80 bp, +52 bp, +76 bp and +95 bp (positions corresponding to the long transcript). Two CAC motifs (CACCC) were detected at -132 bp and +33 bp (Figure 2). Although putative binding sites for transcriptional factors were found *in silico* in pigs, these elements have been confirmed to be required for the transcription of *PGRMC*2 in humans (Bernauer *et al.*, 2001).

The chromosomal location of the porcine *PGRMC*2 gene was determined by IMpRH_7000_ typing. Primers based on exon 1 and intron 1 of the DNA sequence of the porcine *PGRMC*2 gene (Forward: 5'-GGAGATGCTGCTGAAC GTG-3', Reverse: 5'-CTCTCCTGCCCACTACCATC-3') were used to screen the INRA/University of Minnesota porcine radiation hybrid panel (IMpRH_7000_). The PCR results were then run against the IMpRH database of INRA and their anonymous data set (Milan *et al.*, 2000). The retention fraction of *PGRMC*2 was 22%. Two-point analysis revealed that the most significantly linked marker was CL344180 on chromosome 8 at a distance of 19.57cR (LOD score of 10.47). Multipoint analysis showed that the upper and lower markers were CL344180 and CL364915, respectively. The human *PGRMC*2 gene has been assigned to HSA4q26 (UniGene). Comparison of the human and porcine maps showed that SSC8 corresponded entirely to HSA4 p16-q31.3. Hence, our mapping results were consistent with the established conservation of synteny.

The human *PGRMC*2 gene is expressed ubiquitously except in adipose tissue. Gerdes *et al.* (1998) showed that this gene was preferentially expressed in human placenta (UniGene). To determine the expression profile of porcine *PGRMC*2, total RNA was extracted from 18 porcine tissues (adrenal gland, kidney, lung, pituitary, ovary, leaf fat, prostate, testis, heart, thymus gland, epididymis, small intestine, trachea, stomach, liver, hypothalamus, hypothyroid, and urinary bladder) and treated with RNAse-free DNase I (Promega, Madison, WI). The *PGRMC*2-specific RT FP/RP primers are shown in Table 1. The expression of β-actin was used as an internal control under the same conditions. To check for alternative transcripts in other tissues, an additional pair of primers was designed, the forward primer (5'-GTGATGGGGACGTGAAGCTA-3') of which was located in the 5' UTR of the long transcript while the reverse primer (5'-GTCCCGCTTCTTCATACGAG-3') was in the common part of exon 1. A 201-bp *PGRMC*2-specific amplicon was amplified from all 18 tissues, indicating that porcine *PGRMC*2 gene is ubiquitously expressed in pigs (Figure 3). All of the 18 tissues expressed the long transcript of *PGRMC*2. Because the short transcript is included in the long transcript, its expression can not be checked by standard PCR. We were only able to verify the existence of the two *PGRMC*2 splice variants in liver and hypothalamus by RACE-PCR.

**Figure 2:**
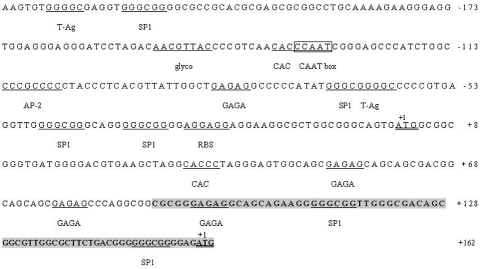
5' genomic DNA sequence of the porcine *PGRMC*2 gene. Important promoter elements including stimulator protein-1 (SP1) binding sites, AP-2 binding site, T-Ag binding sites, GAGA boxes, RBS, the *glyco* box and CAC boxes are underlined. The CCAAT sequence is indicated with a box. The translation initiation site are marked +1. The nucleotide sequence in shaded capitals is the 5' UTR of the short transcript. The sequence was deposited in GenBank under accession no. EU242514.

**Figure 3:**
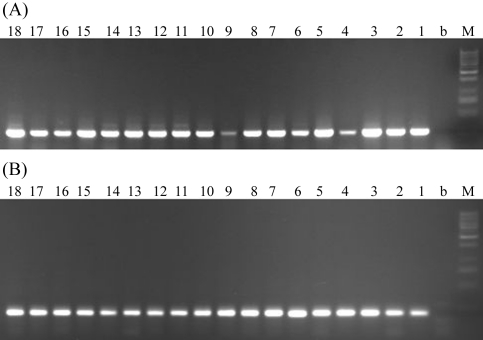
(A) Expression of the porcine *PGRMC*2 gene in different tissues based on RT-PCR. The RNA used for RT-PCR was obtained from the 18 tissues indicated below. The 201-bp PCR products were analyzed on 1.5% agarose gels. Lanes 1-18: 1: adrenal gland, 2: kidney, 3: lung, 4: pituitary, 5: ovary, 6: leaf fat, 7: prostate, 8: testis, 9: heart, 10: thymus gland, 11: epididymis, 12: small intestine, 13: trachea, 14: stomach, 15: liver, 16: hypothalamus, 17: hypothyroid, 18: urinary bladder; b: blank control; m: 1 kb DNA ladder marker; (B) Internal control: porcine β-actin gene.

Single nucleotide polymorphisms (SNPs) in the porcine *PGRMC*2 gene were identified by comparative sequence analysis of the full-length cDNA, part of the introns and the promoter region in samples from two white Duroc boars and two Erhualian sows. Sequences obtained with the primers P2-P9 were used in this analysis. Five SNPs were detected, including a G → A mutation in intron 1, T → G and A → T mutations in intron 2 and two G → A mutations in 3' UTR. There were no SNPs in the coding regions. SNPs in the 3' UTR were reanalyzed by using the software Patrocles Finder to identify potential miRNA targets. A motif (TGCCAAAT) for an unknown miRNA was created by a G > A mutation at EU242513-c.878. The effect of this mutation on translation of the *PGRMC*2 gene and porcine production traits remains to be determined.

## Figures and Tables

**Table 1:** Primer pairs for porcine *PGRMC*2 fragment isolation and SNP identification.

Primers	Primer sequences (5'-3')	Tm (°C)	Product Size (bp)
*PGRMC*2 5' RACE P	F: CAAAATGTCGCCAGTCCTCTGGAG R: Supplied with the BD RACE kit	68	546
*PGRMC*2 5' RACE NP	F: CGAGGCAGAGAAGCGGCTG R: Supplied with the BD RACE kit	60	338
*PGRMC*2 3' RACE P	F: TCGCGGTCAATGGGAAAGTCTTCG R: Supplied with the BD RACE kit	68	1472
*PGRMC*2 3' RACE NP	F: CAACTCTGTCCCCCAACAGC R: Supplied with the BD RACE kit	60	238
P1	F: TTGAATGCCGTACAAATGGA R: ATCTGCAGAGTCCCTTCCAA	59	1234
P2	F: GTCTTCGACGTGACCAAAGG R: TGCATTTCCCATTCTCGAAC	60	14 kb
P3	F: TTGAATGCCGTACAAATGGA R: CCCTGGTTTTAGGAGTCTGC	b	1945
P4	F: GGACAGCGGTTTATGTGACC R: AGCCCACTAAGCCACAAGAG	b	1000
P5	F: GAAGTGTGGGGCGAGGTG R: CCATTGACCGCGAGTAGG	57.6	611
P6	F: GGAGATGCTGCTGAACGTG R: CTCTCCTGCCCACTACCATC	60	649
P7	F: ACCACAATGGGAACTCCAAC R: TAATGACAGCAATGAAAATGG	a	492
P8	F: TGGACCAGGTAAGCAAAAGG R: CCACATCAGTGAGATGTGAG	62	1241
P9	F: AGGAGACCTGGGGAGGAGAG R: CCATTTGGCCATTAACAATG	59	469
*PGRMC*2*- RT FP/RP*	F: TGGATTCTCCCATGCTTCTC R: ATCTGCAGAGTCCCTTCCAA	58	201
β-actin	F : GAGAAGCTCTGCTACGTCGC R : CCAGACAGCACCG TGTTGGC	58	264

a. Five touchdown cycles at 60 °C for 30 s (-1 °C per cycle), followed by 30 cycles at 55 °C for 30 s. b. Five touchdown cycles at 65 °C for 45 s (-1 °C per cycle), followed by 30 cycles at 60 °C for 30 s.
